# Human and pathogen genotype-by-genotype interactions in the light of coevolution theory

**DOI:** 10.1371/journal.pgen.1010685

**Published:** 2023-04-06

**Authors:** Lars Råberg

**Affiliations:** Department of Biology, Lund University, Sweden; Georgia Institute of Technology, UNITED STATES

## Abstract

Antagonistic coevolution (i.e., reciprocal adaptation and counter-adaptation) between hosts and pathogens has long been considered an important driver of genetic variation. However, direct evidence for this is still scarce, especially in vertebrates. The wealth of data on genetics of susceptibility to infectious disease in humans provides an important resource for understanding host–pathogen coevolution, but studies of humans are rarely framed in coevolutionary theory. Here, I review data from human host–pathogen systems to critically assess the evidence for a key assumption of models of host–pathogen coevolution—the presence of host genotype-by-pathogen genotype interactions (G×G). I also attempt to infer whether observed G×G fit best with “gene-for-gene” or “matching allele” models of coevolution. I find that there are several examples of G×G in humans (involving, e.g., *ABO*, *HBB*, *FUT2*, *SLC11A1*, and HLA genes) that fit assumptions of either gene-for-gene or matching allele models. This means that there is potential for coevolution to drive polymorphism also in humans (and presumably other vertebrates), but further studies are required to investigate how widespread this process is.

## Introduction

Population genetic analyses of humans as well as other organisms have shown that immune genes and other genes at the host–pathogen interface are often highly polymorphic. Moreover, many of these polymorphisms are associated with susceptibility to infectious and inflammatory/autoimmune disease and have therefore likely been subject to natural selection [1,2]. Natural selection is normally expected to eliminate genetic variation, so why are immune genes then so variable?

A popular idea is that the high level of polymorphism is a result of host–pathogen coevolution driven by negative frequency-dependent selection (NFDS; Box 1), often referred to as “Red Queen dynamics” [3,4]. This can occur because infection typically requires pathogen binding to some host molecule to gain access to tissue and/or pathogen evasion of immune recognition to avoid clearance. Regardless of the type of molecular interaction, pathogens should evolve to infect common host genotypes that are then selected against and decline in frequency, followed by pathogen adaptation to alternative host genotypes which are then selected against. Such persistent NFDS as a result of continuous pathogen adaptation to the currently most frequent host genotype can lead to the maintenance of 2 or more alternative alleles for long time periods at the loci involved.

**Box 1. Glossary**

Host–pathogen coevolution: a form of antagonistic coevolution, where there is reciprocal selection for adaptation and counter-adaptation in 2 species that affect each other’s fitness negatively.

Negative frequency-dependent selection: when the fitness of an allele is negatively correlated with its frequency (direct NFDS) or the frequency of an allele at another locus (indirect NFDS). In case of host–pathogen coevolution, there needs to be indirect NFDS in the sense that the fitness of a host allele depends on the frequency of the pathogen allele with which it interacts [3,10].

The finding that genes at the host–pathogen interface in humans and other organisms often have signatures of balancing selection [5–7] is clearly consistent with the idea of coevolution by NFDS. However, balancing selection on such genes could also be a result of other forms of pathogen-mediated balancing selection, like heterozygote advantage or spatiotemporal heterogeneity in pathogen abundance driven by environmental factors [3]. None of the latter processes involve reciprocal selection for adaptation and counter-adaptation as in coevolution; instead, they represent unidirectional selection by pathogens on the host.

In invertebrates and plants, “time-shift experiments”—where hosts are exposed to pathogens from the past, present, and future—have demonstrated that coevolution by NFDS indeed plays a role in natural populations [8,9]. However, such experiments are difficult to perform on vertebrates, and there is little other evidence that balancing selection in vertebrates is a result of host–pathogen coevolution by NFDS. Moreover, even if NFDS in principle is a very powerful driver of polymorphism, theoretical models have shown that it only occurs in a quite narrow parameter space [10]. Thus, it is relevant to ask: How important is coevolution, with continuous adaptation and counter-adaptation of host and pathogen, as a driver of polymorphism in vertebrates?

A good way to start investigating the role of coevolution by NFDS in vertebrates is to test assumptions that are specific to models of host–pathogen coevolution. The key assumption of classical models of host–pathogen coevolution by NFDS is that infection depends not only on genetic variation in host and pathogen, but also on the combination of host and pathogen genotypes. Thus, in statistical terms, there needs to be a host genotype-by-pathogen genotype interaction (G×G) for susceptibility to infection [3,4,11].

**Fig 1:**
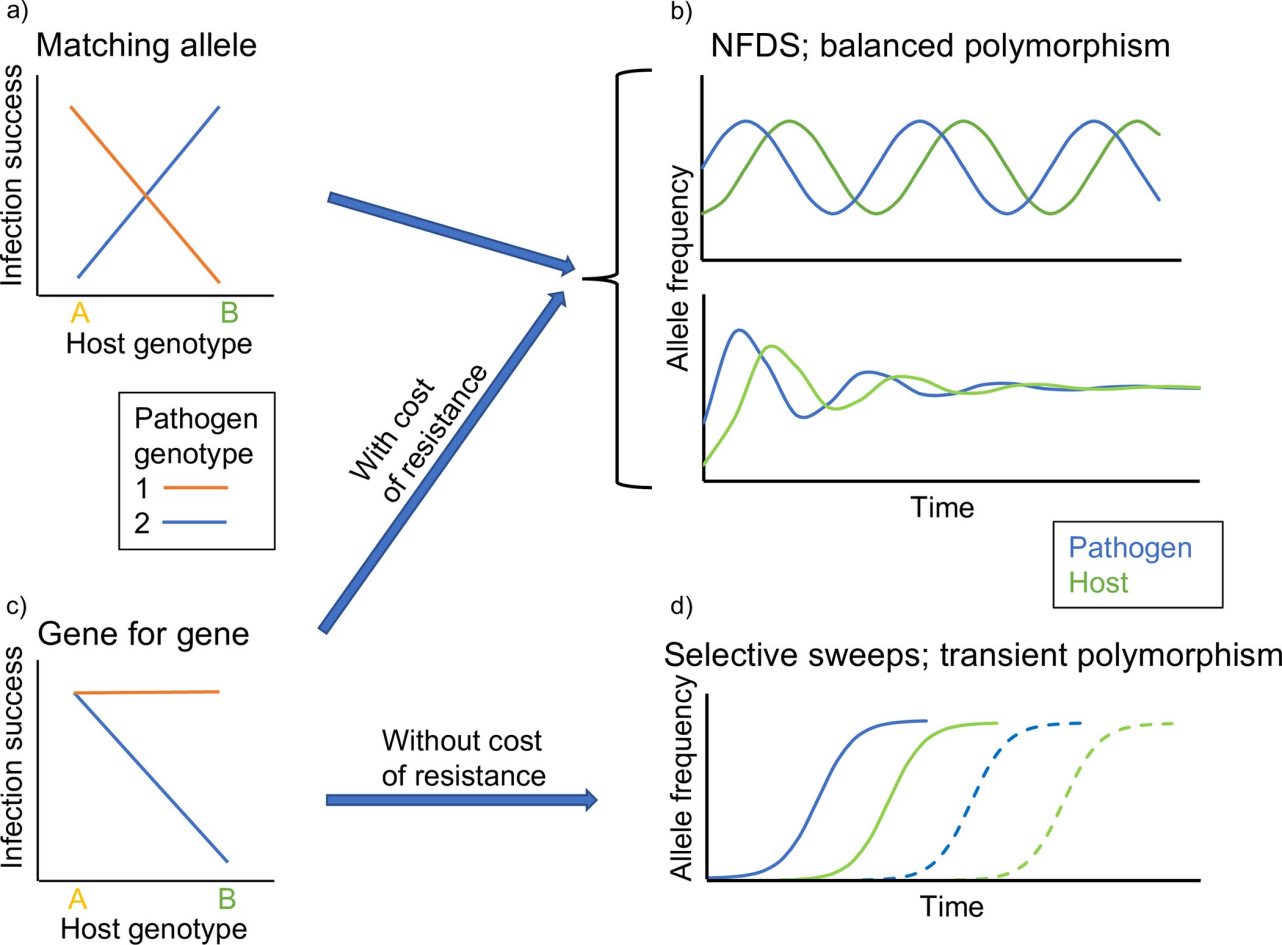
Coevolutionary consequences of different types of G×G. For simplicity, the figure illustrates a scenario where both host and pathogen are haploid and where the G×G involves 1 host locus and 1 pathogen locus (each with 2 different alleles). In MA models, there is a G×G such that different pathogen genotypes infect different host genotypes (a). MA models readily lead to NFDS and the long-term maintenance of polymorphism at interacting loci in both host and pathogen, either in the form of cyclic allele frequencies or a stable polymorphism (b). This occurs because resistance to 1 pathogen genotype comes with a cost in the form of susceptibility to other pathogen genotypes. In other words, under the MA scenario, there is a trade-off between resistance to different pathogen genotypes. In GFG models, there is a G×G such that some pathogen genotypes infect a wider range of genotypes than others (c). In the basic GFG scenario, there is no cost of host resistance or pathogen infectivity. When a host allele that improves resistance without any costs (to the host) occurs in a population, it will be favoured by selection and driven to fixation. Similarly, when a pathogen allele that improves infectivity without costs (to the pathogen) occurs, it will go to fixation. GFG models without costs of resistance or infectivity therefore lead to selective sweeps with only brief, transient polymorphisms, often referred to as arms race coevolution ((d); note that successive sweeps often occur at different sites in the genome, as indicated by different types of lines). However, if there is a cost of host resistance in the currency of another trait related to fitness so that no host genotype has highest fitness under all conditions (and a cost of pathogen infectivity so that no pathogen genotype has highest fitness under all conditions), also GFG models can lead to coevolution by NFDS and the long-term maintenance of polymorphism in the same way as matching allele models (b) [12]. Note that different types of molecular interactions between host and pathogen can result in both MA and GFG type G×G (see [11]). Whether NFDS results in cycles or stable polymorphism (b) depends on the relative importance of 2 different types of NFDS; direct NFDS (where the fitness of an allele is negatively correlated with its frequency) and indirect NFDS (where the fitness of an allele in the host is negatively correlated with the frequency of an allele at the locus involved in G×G in the coevolving pathogen) [10]. Based on figures in [4,11]. GFG, gene-for-gene; G×G, host genotype-by-pathogen genotype interaction; MA, matching allele; NFDS, negative frequency-dependent selection.

There are 2 basic types of models of host–pathogen coevolution, with different types of G×G; “matching allele” (MA) and “gene-for-gene” (GFG) models [10,12]. Briefly, MA models assume G×G such that different host genotypes are susceptible to different pathogen genotypes, while GFG models assume G×G such that host genotypes differ in the range of pathogen genotypes they are susceptible to. Both scenarios can lead to coevolution by NFDS and the long-term maintenance of polymorphism, but the GFG scenario will only do so if there is a cost of resistance (see Fig 1 for details). Thus, testing for G×G and investigating the nature of G×G provides a key to understanding host–pathogen coevolution.

There are numerous studies demonstrating G×G in plant and invertebrate host–pathogen systems (for examples, see [13,14]), but explicit tests for G×G in vertebrates have been scarce [15]. However, during the last decade, several genome-wide tests for G×G in human host–pathogen systems have been published. Here, I systematically review the evidence for G×G from these studies, as well as candidate gene analyses, and evaluate the implications for our understanding of the importance of coevolution between pathogens and humans (and vertebrates in general) as a cause of balancing selection. I focus on the following questions: For which human genes is there evidence of G×G? Are these G×G of MA or GFG type? Are there other types of costs (e.g., risk of autoimmune disease) associated with genes involved in G×G (which could help maintain polymorphism in case of GFG type G×G)? Do genes involved in G×G show signatures of balancing selection (as would be expected if they are engaged in coevolution by NFDS)?

## G×G in humans

### Literature search

Studies of G×G in humans have not used consistent terminology (for example, the term genotype-by-genotype interaction or similar is rarely used in the literature on humans), so it is difficult to perform a focused literature search with narrow search terms. Instead, I identified relevant papers by a combination of broad reading of the literature (particularly review papers of genetics of susceptibility to pathogens in humans) and a literature search with broad search terms (Box 2).

**Box 2. Literature search**

I first identified relevant papers by broad reading of the literature, in particular review papers of genetics of susceptibility to pathogens in humans; this yielded a first set of 13 papers showing G×G, involving 8 different pathogens. To find more papers, I performed a literature search in Web of Science Core Collection in Dec 2022. To this end, I extracted key words from the titles and abstracts of the first set of papers and constructed a query with relatively broad search terms, but which still yielded a manageable number of records [Topic = human AND (genetic varia* OR polymorph*) AND (bacteria* OR viral OR virus OR parasite OR pathogen) AND (interact* OR interplay OR “genome to genome”), which yielded approximately 3.900 records]. By scanning titles and abstracts of these records, I identified papers that considered genetic variation of both host and pathogen; these papers (approximately 1% of the records) were examined in detail (both original results and cited references). In the end, this literature search produced 15 additional papers with evidence for G×G. Most of these concerned pathogens and/or human genes already included in the first set of papers; the list of pathogens and host genes involved in G×G should thus be reasonably complete.

I selected studies showing G×G for any infection-related trait; thus, not only analyses of susceptibility to infection (which is the trait that is traditionally the focus of models of coevolution), but also studies using disease severity, pathogen load, immune escape mutations, etc., as outcome. I only considered natural genetic variation, so studies of genetically modified pathogens or human cell lines were excluded.

### Study types and prevalence of G×G

I found evidence for G×G in 10 human host–pathogen systems, including protozoan, bacterial, and viral pathogens (Table 1). Evidence for G×G comes from several different types of studies, from epidemiological analyses to in vitro assays. Moreover, G×G were detected in several different ways. Several studies tested for G×G between 1 or several host candidate genes and pathogen strains. Another approach, employed in some of the most recent studies, is genome-wide testing for G×G in both host and pathogen, that is by performing genome-wide SNP typing of both host and pathogen and then testing for G×G between all pairs of host and pathogen SNPs, referred to as “genome-to-genome” analysis [16]. Other studies used various combinations of candidate gene analysis, pathogen strain identification, and genome-wide analyses.

To gain insight into how common G×G are it is useful to focus on studies based on genome-wide analyses of humans (genome-to-genome studies and genome-wide tests for interactions with pathogen strains), as they should provide a more unbiased estimate of the occurrence of G×G than candidate gene studies. Genome-wide analyses have been performed with viral and bacterial pathogens. All 3 genome-wide tests for G×G with viruses found evidence for G×G [17–19]. Most of these concern immune escape mutations, the only exception being [18], which also found G×G for viral load. Of the genome-wide analyses for G×G with bacterial pathogens, 2 studies found statistically significant G×G [20,21] while 1 did not [22]. In all studies where G×G were found, only 1 or a few host loci were involved. Thus, the currently available data indicate that G×G occur in most host–pathogen pairs, but that at most a few host genes are involved in each pair. It should be noted, though, that the multiple testing burden is considerable in genome-wide tests for G×G [16], so future studies with higher power may reveal that a larger number of host loci are often involved in G×G in each host–pathogen pair.

**Table 1:** Evidence for G×G in human host–pathogen systems.

Pathogen	Human gene	Pathogen *gene*/protein	Type of study	G x G test (host–pathogen)	Trait	Effect of host polymorphism for each pathogen genotype	GFG/MA^1^	Signature of balancing selection?	Cost of resistance?
*Plasmodium falciparum*	*HLA-B*	*csp*	Naturally infected (cases)	Candidate gene x candidate gene	Clinical malaria	HLA-B*35 associated with higher frequency of *2 P*. *falciparum* genotypes and lower frequency of 2 genotypes [53].	?	Yes [5,54].	Yes; the haplotype involved in G x G (HLA-B x 35) is associated with a number of diseases, with OR both >1 and <1 [40].
*Plasmodium falciparum*	*HBB*	*msp-1*	Naturally infected (cases)	Candidate gene x candidate gene	*P*. *falciparum* infection	Frequencies of *msp-1* genotypes differ between *HBB* AS and AA individuals [55].	?	Yes; recent positive/balancing selection [47].	Yes; HbS leads to anemia and associated diseases [56].
*Plasmodium falciparum*	*HBB*	3 genes on 2 chromosomes; all in strong LD	Case-control	Candidate genes x genome wide	Severe malaria	HbS protective against severe malaria if infected with parasite having major alleles at all 3 genes (OR≈0.02) but not when infected with parasite having minor alleles (OR≈1) [23].	GFG	Yes; recent positive/balancing selection [47].	Yes; HbS leads to anemia and associated diseases [56].
*Streptococcus pneumoniae*	*SASH1*, *FARP1*	*pspC*	Case-control	Genome x candidate genes	Pneumococcal meningitis	*SASH1*: OR for 1 pspC allele >1; OR for 2 other pspC alleles not ≠ 1. *FARP1*: OR for 1 pspC allele >1; OR for 2 other pspC alleles not ≠ 1 [20].	GFG	No	No known.
*Streptococcus pneumoniae*	*STK32C*		Case-control	Genome x pathogen strain (”serotype”)	Pneumococcal meningitis	OR for 1 serotype > 1; OR for 6 other serotypes not ≠ 1 [20].	GFG	No	No known.
*Mycobacterium tuberculosis*	*HLA-DRB1*, *HLA-DQB1*		Case-control	Candidate gene x pathogen strain	Tuberculosis	DRB1*09:*01* OR and DQB1*03:*03* OR for “modern strains” >1; OR for other strains not ≠ 1 [26]	GFG	Yes [5,54]	Yes [40].
*Mycobacterium tuberculosis*	*HLA-A*, *HLA-B*, *HLA-C*		Naturally infected (cases)	Candidate gene x pathogen strain	Pulmonary tuberculosis	Specific HLA class I alleles associated with specific *M*. *mycobacterium* strains [57].	?	Yes [5,54]	Yes [40].
*Mycobacterium tuberculosis*	*TLR2*		Case-control	Candidate gene x pathogen strain (“lineage”)	Tuberculous meningitis	OR for Beijing lineage >1; OR for other lineages not ≠ 1 [24].	GFG	No	No known.
*Mycobacterium tuberculosis*	*CD209*		Naturally infected (cases)	Candidate gene x pathogen strain	Mortality from pulmonary tuberculosis	Infection with Beijing vs. other strains in patients who died from TB associated with SNP in *CD209* [58].	?	No	No known.
*Mycobacterium tuberculosis*	*SLC11A1*		Naturally infected (cases)	Candidate gene x pathogen strain	Pulmonary tuberculosis	Infection with Beijing vs. other strains associated with 2 SNPs in *SLC11A1* [59].	?	(Yes) [60].	Yes; see below.
*Mycobacterium tuberculosis*	*SLC11A1*		Naturally infected (cases)	Candidate genes x pathogen strain (“lineage”)	Tuberculosis severity	Opposite effects of *SLC11A1* alleles in individuals with lineage “L4.6 Uganda” vs. other lineages [35].	MA	(Yes) [60].	Yes; high expressing allele associated with autoimmune diseases while low expressing allele associated with infectious diseases [44].
*Mycobacterium tuberculosis*	*PPIAP22*		Naturally infected (cases)	Genome x pathogen strain (“lineage”)	Tuberculosis severity	Opposite effects of *PPIAP22* alleles on disease severity in individuals with lineage “L4.6 Uganda” vs. other lineages [21].	MA	No	No known.
*Helicobacter pylori*	*ABO*	*babA*	In vitro functional analysis	Candidate gene x pathogen strain (“isolate”)	Binding of host receptor	Most strains bind both A and H antigen (generalists), but significant fraction of strains in S America are specialists and bind only H (i.e., blood group O) [29].	Mainly GFG	Yes [46].	Yes; blood group A, B, and AB increase susceptibility to severe malaria [27,61].
*Vibrio cholerae*	*ABO*	*ctxAB*	Case-control	Candidate gene x pathogen strain (“serogroup”/“biotype”)	Disease severity	Blood group O confers higher risk of severe disease than blood group A or B, but only when infected with O1 El Tor or O139 strains; in contrast, no association between disease severity and ABO group for classical O1 strains [27].	GFG	Yes [46].	Yes; blood group A, B, and AB increase susceptibility to severe malaria [27,61].
HIV	*HLA-A*, *HLA-B*, *HLA-C*	48 amino acid residues throughout HIV proteome	Naturally infected (cases)	Genome x genome	Immune escape mutations	48 HIV-1 amino acid variants associated with SNPs in *HLA-A*, *B*, or *C* [17]. See also [30].	?	Yes [5,54].	Yes [40].
HIV	*HLA-A*, *HLA-B*, *HLA-C*	Protease/RT, Nef, Vpr	Naturally infected (cases)	Candidate genes x candidate genes	Immune escape mutations	Different *HLA-A*, *HLA-B*, and *HLA-C* alleles often select for different amino acid escape mutations at a given HIV residue (at 23/57 residues where polymorphism is associated with specific HLA alleles) [31].	MA for at least 23/57 HIV residues involved in GxG.	Yes [5,54].	Yes [40].
HIV	*HLA-A*, *HLA-B*, *HLA-C*	31 amino acid residues throughout HIV proteome	Naturally infected (cases)	Candidate genes x genome	Immune escape mutations and viral load	Specific *HLA-A*, *HLA-B*, and *HLA-C* alleles select for specific amino acid escape mutations at 31 HIV residues (but no case where different *HLA* alleles select for different amino acids at a given residue, as in [31]). In addition, effect of *HLA-A/B* allele x immune escape mutation on viral load at 3 of the 31 residues, such that viral load is reduced in individuals carrying specific *HLA* alleles if HIV has not acquired escape mutation [32].	Immune escape:?; viral load: GFG.	Yes [5,54].	
HIV	*KIR2DL2*	Vpu, Env	In vitro infection assay	Candidate gene x candidate gene	Viral replication	NK cells with ≥1 copy of *KIR2DL2* inhibit replication of HIV Vpu-Env(WT/WT) but not Vpu-Env (V/V), but NK cells w/o *KIR2DL2* inhibit neither HIV genotype [34].	GFG	Yes [62].	Yes; haplotype w KIR2DL2 associated w several AID, including T1D and UC [43].
Hepatitis C virus	*HLA-A*, *HLA-B*, *HLA-C*, *DQA1*, *DRB1*	NS3, NS4B	Naturally infected (cases)	Genome x genome	Immune escape mutations	Specific HLA alleles select for specific amino acid escape mutations at certain residues, but no case where different *HLA* alleles select for different amino acids at a given residue [18]. See also [33].	?	Yes [5,54].	Yes [40].
Hepatitis C virus	*IFNL4*	NS5B	Naturally infected (cases)	Genome x genome	Immune escape mutations and viral load	Immune escape: *IFNL4* rs12979860 associated with amino acid variants at 11 HCV residues. Viral load: *IFNL4* rs12979860 C>T associated with reduced viral load, but only if HCV has serine at site 2414 [18]. See also [63].	Immune escape:?; Viral load: GFG	No (but population-specific positive selection; [64]).	No known.
Hepatitis C virus	*KIR* genes		Naturally infected (cases)	Candidate gene x pathogen strain (HCV genotype)	Risk of hepatocellular carcinoma (HCC)	For HCV genotype 1, 2, and 3, risk of HCC decreases with number of activating *KIR* genes; for HCV genotype 4, risk is low regardless of number of activating *KIR* genes [36].	GFG	Yes [62].	Yes, several activating *KIR* genes associated with autoimmune diseases [43].
Norovirus	*FUT2*		Challenge studies/case-control/prospective cohort studies	Candidate gene x pathogen strain	Acute gastroenteritis	Challenge studies: Nonfunctional *FUT2* protects against strain GI.1 and GII.4, but not against GII.2. Case-control studies: Nonfunctional *FUT2* protects against GII.4, but not against GI.3. Prospective cohort studies: Nonfunctional *FUT2* protects against GII.3 and GII.4, but not against GI.3, GI.6, GII.1, GII.2, and GII.7 [25].	GFG	Yes [65,66].	Yes; nonfunctional *FUT2* associated w susceptibility to Crohn’s disease [41] and other diseases [42].
Epstein-Barr virus (EBV)	*UNC5D*, *LINC01830*, non-coding region on chr 7	BALF5, BBRF1, BRLF1	Naturally infected (cases)	Genome x genome	Immune escape mutations	25 host SNPs in 3 genomic regions (17, 1, and 7 SNPs, respectively) associated with variants in 3 EBV genes [19].	?	Yes, *UNC5D* [67].	Possibly for *UNC5D;* SNP in this gene associated with adolescent idiopathic scoliosis.
Epstein-Barr virus (EBV)	*HLA-B*	EBNA-1	In vitro functional analysis	Candidate gene x pathogen strain	In vitro immune response	EBV peptide variant HPVG most immunogenic on HLA-B*35:01, but peptide variant HPVG-D5 most immunogenic on HLA-B*35:08 [37].	MA	Yes [5,54].	Yes [40].
Human papillomavirus (HPV)	*HLA-DR/DQ*		Case-control	Candidate gene x pathogen strain	Cervical cancer	Different *HLA-DR-DQ* haplotypes affect resistance/susceptibility to cancer caused by different HPV types [28].	MA	Yes [5,54].	Yes [40].
Human papillomavirus (HPV)	*HLA-DR/DQ*	E6	Naturally infected (cases; i.e., cervical cancer patients)	Candidate gene x candidate gene	Infection (or immune escape mutation?)	*HLA-DR04-DQ03* haplotype associated with HPV E6 L83V variant in cervical cancer patients [68,69].	?	Yes [5,54].	Yes [40].

^1^ GFG = gene-for-gene type G×G, MA = matching allele type G×G,? = type of G×G could not be inferred from published data. For case-control studies of binary traits (presence/absence of infection or disease), GFG was inferred when the OR for ≥1 pathogen genotype was different from 1 while the OR for ≥1 other pathogen genotype was equal to 1, whereas MA was inferred when the OR for different pathogen genotypes where in opposite directions. For analyses of continuous traits (pathogen load, disease severity), GFG was inferred when there was an effect of host genotype on the trait for ≥1 pathogen genotype but no effect of host genotype for other pathogen genotypes, whereas MA was inferred when host genotype had opposite effects on the trait for different pathogen genotypes. For escape mutations, MA was inferred when alternative alleles at a HLA gene were associated with different escape mutations at a given pathogen residue (see main text for more detailed explanations).

### For which genes is there evidence of G×G?

Human genes with evidence for G×G include some genes that are textbook examples of associations with susceptibility to infectious disease, such as the MHC class I genes (*HLA-A*, *HLA-B*, *HLA-C*; G×G with, e.g., *Plasmodium falciparum* and HIV) and the blood group antigen gene *ABO* (G×G with *Helicobacter pylori* and *Vibrio cholerae*). A recent study also found G×G between *HBB* (encoding the hemoglobin β subunit)—which has well-known effects on susceptibility to malaria—and *P*. *falciparum* [23]. Specifically, the protective effect of the HbS variant at *HBB* was found to depend on the genotype at 3 different loci in the *P*. *falciparum* genome, with all 3 loci in strong linkage disequilibrium such that the minor alleles occur together.

In addition, genes with evidence for G×G include some canonical immune genes (e.g., *TLR2*, *IFNL4*, *KIR2DL2*, *CD209*) and other genes at the host–pathogen interface with well-documented associations with susceptibility to infection (*FUT2*, *SLC11A1*), but also several genes that are not previously recognised in this context (e.g., *FARP1*, *STK32C*, *UNC5D*).

### Are these G×G of MA or GFG type?

None of the studies put their results in the context of MA versus GFG. I therefore inferred the type of G×G based on the published data. G×G were detected for several different infection phenotypes, including both binary (e.g., infection status) and continuous traits (e.g., pathogen load or disease severity in infected individuals). For case-control studies of infection status or other binary disease phenotypes, where it is possible to calculate host genotype odds ratios (OR) separately for each pathogen genotype, this information can be used to distinguish MA and GFG. To see this, consider the simplest case where there is G×G between a pair of loci that are bi-allelic in both host and pathogen, as in Fig 1. If there is a trade-off between resistance to different host genotypes as in the MA scenario (Fig 1A), host genotype should be associated with both pathogen genotypes, but in opposite ways. Thus, the OR should be >1 for one pathogen genotype but <1 for the other (note that none of the ORs need to be significantly different from 1, but they should be different from each other). In contrast, if host genotypes differ in the range of pathogen genotypes they are resistant/susceptible to, as in the GFG scenario (Fig 1C), host genotype should only be associated with one of the pathogen genotypes. Thus, the OR should be significantly different from 1 for one pathogen genotype but equal to 1 for the other.

Of the case-control studies with evidence for G×G for infection status or other binary disease phenotypes, 8 present pathogen genotype-specific ORs, with 1 to 3 host genes involved in G×G with each pathogen [20,23–28]. In all but 1 case, the OR is significantly different from 1 for one pathogen genotype but not for others. Thus, in the majority of cases the pattern is most consistent with GFG type G×G. Perhaps the most striking example is *HBB* and resistance to malaria [23]. Here, HbS is protective against severe malaria if an individual is infected with a parasite having the major allele at all 3 loci involved in G×G (OR≈0.02) but not when infected with parasites having the minor allele at all 3 loci (OR≈1) (based on data in Fig 2 of [23]). Similar differences between host genotypes in the range of pathogen strains they are susceptible to (but without any indication of a trade-off between resistance to different strains) are seen with for example *ABO* (with *H*. *pylori* and *V*. *cholera*) [27,29] and *FUT2* (with Norovirus) [25]. The only indication of MA type G×G in case-control studies concern HLA class II and risk of cervical cancer caused by human papilloma virus (HPV), where different HLA haplotypes affect susceptibility to different HPV types [28].

**Fig 2:**
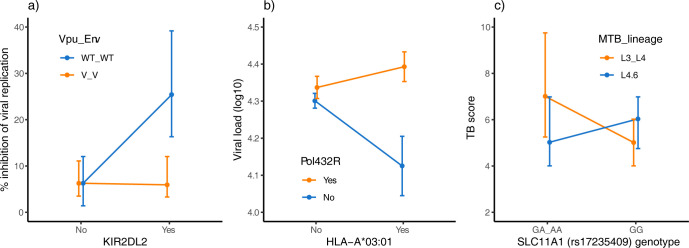
Examples of G×G for continuous traits related to resistance/susceptibility to infectious disease. (a) G×G between NK cell Killer Immunoglobulin Receptor genotype and HIV genotype for inhibition of viral replication (median ± interquartile range). NK cells with the *KIR2DL2* allele strongly inhibit replication of HIV with wild-type alleles at *vpu* and *env* (WT_WT) but have limited inhibitory effect on HIV with variant alleles (V_V), while NK cells without *KIR2DL2* have limited inhibitory effect on both WT_WT and V_V. Thus, presence/absence of *KIR2DL2* affects the range of HIV genotypes an individual is susceptible to, consistent with the GFG scenario. Data extracted from Fig 1B (day 3) of [34]. (b) G×G between *HLA-A* genotype and HIV genotype for viral load (mean ± SE). Arginine (R) at residue 432 in the *pol* gene is an immune escape mutation from the *HLA-A* allele 03:01. In individuals carrying A*03:01, viral load is suppressed in infections with virus without the Pol432R escape mutation, while there is no effect of Pol432 genotype on viral load in individuals without A*03:01 (and no G×G for viral load between Pol432 genotype and other HLA alleles). Thus, there is no indication of a trade-off between resistance to Pol432R and other genotypes as would be the case in an MA type G×G; instead the pattern is consistent with a GFG type G×G. Data from [32]. (c) G×G between *SLC11A1* genotype and *M*. *tuberculosis* lineage for tuberculosis severity (median ± IQR). A recently evolved *M*. *tuberculosis* sublineage (L4.6) in combination with homozygosity for an ancestral *SLC11A1* allele (genotype GG) and an original *M*. *tuberculosis* lineage (L3 or L4) in combination with ≥1 derived *SLCA11A1* allele (genotype GA or AA) is associated with more severe disease than the other combinations of host and pathogen genotypes (the interaction is highly significant: *P =* 0.00022). Thus, there is a trade-off between resistance to disease by different lineages, consistent with an MA type G×G. Data extracted from Fig 2 in [35]. GFG, gene-for-gene; G×G, host genotype-by-pathogen genotype interaction; MA, matching allele.

There are also several studies that have performed analyses of associations between pathogen and host alleles in chronic viral infections [17–19,30–33]. Such G×G are generally interpreted as being a result of within-host evolution of immune escape, although they could also reflect differences in susceptibility to infection with viruses carrying different alleles at the start of the infection. These studies have primarily found G×G involving HLA genes. It is generally difficult to infer whether these G×G are of MA or GFG type, because specific HLA alleles are often associated with escape mutations at several positions in the viral genome. However, one of the studies of HIV found that different HLA alleles were associated with different amino acid escape mutations at a particular position [31], a pattern clearly indicating a trade-off between resistance to different pathogen genotypes; thus in at least some cases G×G for escape mutations are consistent with the MA scenario.

Besides studies based on epidemiological analyses of presence/absence of infectious disease or immune escape mutations, there are also some studies finding G×G for various continuous infection-related traits like pathogen replication and disease severity [18,21,32,34–37]. Given that the trait is associated with both host and pathogen fitness, also G×G affecting such traits could lead to coevolution. A study using an in vitro assay of HIV replication found that NK cells with at least 1 copy of the *KIR2DL2* allele inhibit replication of 1 specific HIV genotype, while NK cells without *KIR2DL2* have limited inhibitory effect regardless of HIV genotype [34], a pattern consistent with a GFG type G×G (Fig 2A). Similarly, a study analysing effects of HIV escape mutations on viral load showed that certain virus genotypes had reduced viral load in individuals carrying a specific MHC allele while there was no effect of virus genotype on viral load in individuals without that allele; also this pattern appear consistent with the GFG scenario (Fig 2B) [32]. In contrast, an analysis of tuberculosis patients showed that *SLC11A1* genotype had opposite effects on disease severity depending on *Mycobacterium tuberculosis* lineage [35], consistent with an MA type G×G (Fig 2C). Overall, 4 of the 7 analyses of continuous traits showed results consistent with GFG type G×G, while 3 are consistent with MA type G×G (Table 1).

Finally, in vitro functional analyses of the ability of different *Helicobacter pylori* isolates to bind host receptors showed that most isolates are generalists and bind both A and H antigen (from individuals with blood group A and O, respectively) while a significant fraction of strains in South America are specialists and bind only H antigen [29], consistent with GFG type G×G.

### Are there other types of costs associated with genes involved in G×G?

Since several of the G×G appeared to be of the GFG type, it would be of interest to know if the genes involved are associated with other types of diseases that could lead to the fitness cost necessary to generate NFDS and help maintain polymorphism in case of a GFG type G×G (Fig 1). To identify potential costs of alleles conferring resistance to a particular pathogen genotype, I searched the GWAS catalog [38] (either directly or via LDtrait at LDlink [39] to check if SNPs involved in G×G were in linkage disequilibrium with SNPs associated with other diseases) and PheWAS Resources (*HLA* genes) [40] for disease-associations with genes involved in G×G.

For several of the genes with GFG type G×G, there is indeed strong evidence for costs associated with the allele that confers resistance to a subset of pathogen genotypes. For example, the nonfunctional *FUT2* allele, which protects against some Norovirus strains, is also associated with Crohn’s disease and other diseases [41,42]. Similarly, *KIR2DL2*, which inhibits replication of a specific HIV genotype [34], is associated with several autoimmune diseases [43]. Overall, costs are known for about two thirds of the genes with indication of GFG type G×G (Table 1). Interestingly, there is also evidence for costs of resistance in case of *SLC11A1*, which is one of few genes showing clear MA type G×G (where costs are not necessary to generate NFDS; Fig 1). Here, high and low expression alleles are associated with susceptibility to autoimmune and infectious disease, respectively [44].

### Do genes involved in G×G show signatures of balancing selection?

If the G×G identified in humans really lead to coevolution by NFDS, one would expect the genes involved to exhibit signatures of balancing selection that can be detected by analyses of population samples of DNA sequence data [45]. For 12 of the 20 genes involved in G×G, there are such signatures of balancing selection, based on genome-wide scans or candidate gene analyses (Table 1). Most show signatures of long-term balancing selection, in some cases—for example, *ABO—*in the form of “trans-species polymorphisms,” meaning that the polymorphism has been maintained by selection in primates for tens of millions of years [46]. An exception to the trend for long-term balancing selection is *HBB* that shows a signature of recent positive or balancing selection (for recent selection the signatures of positive and balancing selection are indistinguishable) [47].

## Conclusions

The present review has shown that several human genes are involved in G×G, as assumed by models of host–pathogen coevolution. Most of the G×G seem to fit the GFG rather than MA scenario, particularly for case-control studies of infection status and other binary disease phenotypes, which means a cost of resistance is required for these G×G to lead to maintenance of polymorphism by NFDS. Such costs are known for at least some of the genes with evidence for G×G. Taken together, this shows there is scope for coevolution by NFDS also in vertebrates. These conclusions come with several caveats, though.

First, for G×G to result in coevolution, the phenotypic trait concerned must be associated with both host and pathogen fitness. While most studied traits (Table 1) clearly can affect host fitness, the relevance for pathogen fitness is doubtful in some cases, for example, meningitis in *Streptococcus pneumoniae* infection [20] and risk of cervical cancer caused by HPV [28]. Second, in case of chronic viral infections (HIV, HCV, and EBV), the G×G are thought to be a result of within-host evolution of immune escape, and it is not always clear if these G×G also affect some aspect of host fitness, such as susceptibility to infection or severity of disease, as would be required for coevolution to occur. However, a recent study of HIV found that at least some of the immune escape mutations led to G×G for viral load [32], indicating that G×G involving immune escape mutations might indeed affect host fitness. Third, inferring if G×G are of GFG or MA type from currently segregating host and pathogen alleles might be misleading. For example, what is actually an MA type G×G might appear to be a GFG type G×G if rare alleles are not sampled [11,48]. Fourth, the preponderance of GFG type G×G in case-control studies of binary disease phenotypes might be an artefact of that these analyses are based on separate analyses of each pathogen strain and only report host polymorphisms where the OR is different from 1 for at least one of the pathogen strains. Thus, these analyses will miss MA type G×G where the OR for 2 pathogen strains are in opposite directions and different from each other, but none is different from 1. Even with these caveats in mind, there are some strong cases for coevolutionarily relevant G×G of both GFG and MA type (GFG: e.g., *HBB*, *ABO*, *FUT2*, and HLA genes; MA: e.g., *SLC11A1* and HLA genes).

The G×G for *HBB* illustrates that different types of pathogen-mediated balancing selection can act on a given gene simultaneously. *HBB* is the textbook example of heterozygote advantage, where individuals with 1 copy of the HbS variant have improved resistance to malaria, whereas HbS homozygosity leads sickle cell disease [49]. The finding that *HBB* is involved in a G×G with *P*. *falciparum* shows that there might also be NFDS on this gene. It is often expected that several different types of pathogen-mediated balancing selection operate simultaneously on a given gene and *HBB* is perhaps the clearest evidence yet that this is the case.

The G×G for *ABO* and the HLA genes illustrate another aspect of pathogen-mediated balancing selection—that a given gene might be coevolving with more than 1 pathogen simultaneously, so called “diffuse coevolution” [3]. Diffuse coevolution is expected to be common, perhaps the norm, but *ABO* and the HLA genes are as far as I am aware the first cases where specific genes have been shown to be involved in G×G with 2 or more different pathogens, thus demonstrating that there actually is opportunity for diffuse coevolution.

In conclusion, there is some evidence from humans for G×G, a key assumption of models of host–pathogen coevolution by NFDS (but not other types of pathogen-mediated balancing selection). This indicates that balancing selection on genes at the host–pathogen interface in humans (and other vertebrates) could indeed be a result of coevolution, as is commonly assumed. Nevertheless, more studies testing for G×G are clearly desirable, in particular, genome-to-genome studies as they give an unbiased perspective on which genes are involved. Recent development of statistical approaches should facilitate this [50,51]. Still, it is important to recognise that the presence of G×G only shows that there is opportunity for coevolution by NFDS, not that it has occurred. Demonstrating that polymorphism is a result of coevolution would require additional analyses. One way would be to test if there is NFDS. Specifically, balancing selection by antagonistic coevolution requires indirect NFDS, i.e., the fitness of a host allele should be negatively correlated with the frequency of a pathogen allele. This could be tested by following 1 or more populations over time. Advances in the analysis of ancient DNA from both mammals and pathogens should make this possible even for humans and other species with long generations times [52]. Nevertheless, identifying the genes involved in G×G—as described in this review—would be a critical first step.
